# Evaluation of the Effects of Mesoglycan on Some Markers of Endothelial Damage and Walking Distance in Diabetic Patients with Peripheral Arterial Disease

**DOI:** 10.3390/ijms18030572

**Published:** 2017-03-06

**Authors:** Derosa Giuseppe, D’Angelo Angela, Davide Romano, Maffioli Pamela

**Affiliations:** 1 Centre of Diabetes and Metabolic Diseases, Department of Internal Medicine and Therapeutics, University of Pavia and Fondazione IRCCS Policlinico San Matteo, 27100 Pavia, Italy; labmedmol@smatteo.pv.it (D.A.); dr.davideromano85@gmail.com (D.R.); pamelamaffioli@hotmail.it (M.P.); 2 Centre for the Study of Endocrine-Metabolic Pathophysiology and Clinical Research, University of Pavia, 27100 Pavia, Italy; 3 Centre for Prevention, Surveillance, Diagnosis and Treatment of Rare Diseases, Fondazione IRCCS Policlinico San Matteo, 27100 Pavia, Italy; 4 Laboratory of Molecular Medicine, University of Pavia, 27100 Pavia, Italy

**Keywords:** endothelial damage, mesoglycan, peripheral artery disease

## Abstract

The aim of this study was to evaluate the variation of some parameters involved in peripheral artery disease progression in diabetic patients with peripheral artery disease after six months of mesoglycan. We enrolled 64 Caucasian, type 2 diabetic patients, with stage IIa peripheral artery disease. They were randomized to mesoglycan (Prisma^®^), 50 mg twice a day, or placebo, for six months. We evaluated: glycemic control, metalloproteinase-2, and -9 (MMP-2, and -9), soluble intercellular adhesion molecule-1 (sICAM-1), soluble vascular cell adhesion protein-1 (sVCAM-1), interleukin-6 (IL-6), soluble E-selectin (sE-selectin), high sensitivity C-reactive protein (hs-CRP), tumor necrosis factor-α (TNF-α), and plasminogen activator inhibitor-1 (PAI-1). We recorded a decrease of MMP-2, MMP-9, sE-selectin, TNF-α, sVCAM-1, and IL-6 compared to baseline, and to placebo in the group treated with mesoglycan. Regarding sICAM-1, and hs-CRP, instead, we recorded a decrease with mesoglycan only compared to baseline. Preliminary results seem to suggest an improvement of pain free walking distance with mesoglycan in 18 patients both compared to baseline and to placebo, even if data should be taken cautiously. Our study showed that supplementation with mesoglycan improved endothelial dysfunction in type 2 diabetic patients with peripheral artery disease. Regarding the preliminary data suggesting also a slight improvement of clinical parameters such as pain free walking distance, more data and a bigger sample of patients are necessary to better verify this aspect.

## 1. Introduction

Glycosaminoglycans are essential components of endothelial and vascular walls that are involved in different biological functions [1]. Mesoglycan, a natural glycosaminoglycans preparation, is a polysaccharide complex rich in sulphur radicals with strong negative electric charge. It is extracted from porcine intestinal mucosa and is composed by heparan sulfate, dermatan sulfate, electrophoretically slow-moving heparin, and chondroitin sulfate [2]. Data on mesoglycan showed anti-thrombotic and profibrinolytic activities of the drug; for this reason, mesoglycan may be useful in the management of vascular diseases, when combined with anti-thrombotics, and vasodilator drugs in patients with chronic peripheral arterial disease [3,4,5]. Mesoglycan can have an anti-atherogenic effect with the inhibition of platelet adhesion, stimulation of lipoprotein lipase enzyme, inhibition of smooth muscle cells proliferation; and/or anti-thrombotic effects with activation of anti-thrombin III and heparin cofactor II, and profibrinolytic action (tissue plasminogen activator stimulation). These effects are due to the presence of heparan and dermatan sulfate, fundamental constituents of the vessel wall. In addition to anti-thrombotic effects, mesoglycan is able to restore the physiological properties of selective barriers of capillary endothelium with an anti-edema activity. In humans, the administration of mesoglycan gave a reduction of the number of micro-hemorrhages, microaneurysms, and exudates in patients suffering from diabetic retinopathy [6]. Chronic peripheral arterial disease (PAD) is a clinical syndrome related to the reduction of blood flow to the lower limbs. The main symptom of PAD is intermittent claudication, defined as a cramping pain in the lower limb muscles that occurs after the subject has walked a certain distance; pain stops after the patient rests for a while. The pain occurs every time at the same effort and recedes promptly with the termination of the effort level that triggered it. 

The aim of our study is to evaluate the variation of some of the parameters used to evaluate peripheral artery disease progression, and the change of certain inflammatory parameters in diabetic patients with peripheral artery disease after six months of mesoglycan intake compared to placebo.

## 2. Results

### 2.1. Study Sample

We enrolled 64 patients; 33 were randomized to mesoglycan and 31 to placebo (Table 1 and Table 2). 59 patients completed the study. Five patients did not complete the study and the reasons for prematurely withdrawal included: lost to follow-up (two patients), lack of compliance to treatment (two patients), withdrawn of consent (one patient). Concomitant medication taken at baseline are listed in Table 3.

### 2.2. Glycemic Control

No variations of glycemic control were recorded with mesoglycan or placebo (Table 1). 

### 2.3. Endothelial Damage Markers

**Figure 1:**
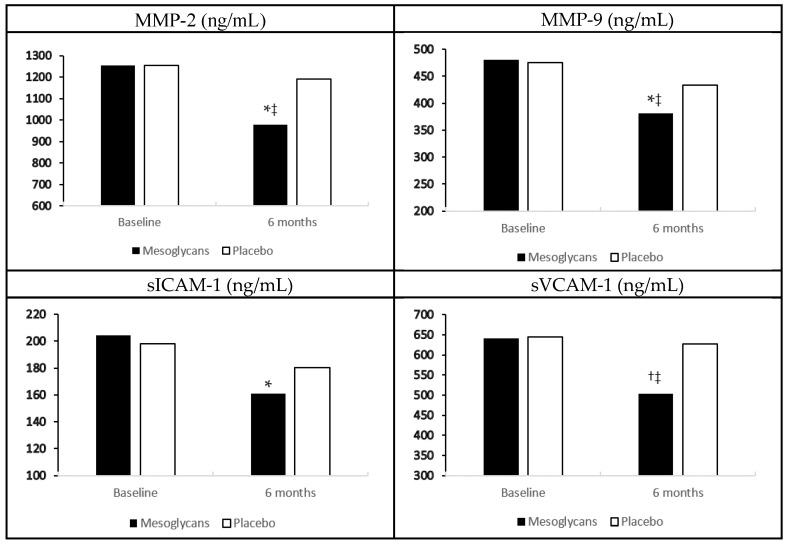
Endothelial damage biomarkers variation with mesoglycan and placebo. Data are expressed as mean ± standard deviation; * *p* < 0.05 vs. baseline; ^†^ *p* < 0.01 vs. baseline; ^‡^ *p* < 0.05 vs. placebo.

In the group treated with mesoglycan, we recorded a decrease of metalloproteinase-2 (MMP-2), and metalloproteinase-2 (MMP-9), soluble E-selectin (sE-selectin), and tumor necrosis factor-α (TNF-α) compared to baseline (*p* < 0.05 for all), and to placebo (*p* < 0.05 for all). We also observed a reduction of soluble vascular cell adhesion protein-1 (sVCAM-1) and interleukin-6 (IL-6), both compared to baseline (*p* < 0.01), and to placebo (*p* < 0.05) with mesoglycan. Regarding soluble intercellular adhesion molecule-1 (sICAM-1) and high sensitivity C-reactive protein (hs-CRP), instead, we recorded a decrease with mesoglycan compared to baseline (*p* < 0.05), but not compared to placebo. No variations of these markers were record with placebo (Figure 1, Table 1). 

### 2.4. Pain Free Walking Distance and Ankle/Brachial Index

With the limitation of the small sample considered, we recorded an improvement of pain free walking distance with mesoglycan in 18 patients both compared to baseline and to placebo (*p* < 0.01 vs. baseline, and *p* < 0.05 vs. placebo). The majority of these 18 patients reported no symptoms during daily activities, even if this data is limited by the fact that the symptomless was self-reported.

Ankle/brachial index was increased with mesoglycan in 18 patients, but not by placebo, both compared to baseline (*p* < 0.05), and to the other group (*p* < 0.05) (Table 2). 

### 2.5. Transcutaneous Oxygen Pressure

Transcutaneous oxygen pressure increased with mesoglycan in 18 patients, both compared to baseline and to placebo (*p* < 0.05 for both). No variations were recorded with placebo (Table 2).

## 3. Discussion

As the major regulator of vascular homeostasis, the endothelium exerts a number of vasoprotective effects, such as vasodilation, suppression of smooth muscle cell growth, and inhibition of inflammatory responses. Accumulating evidence suggests that endothelial dysfunction is an early marker for atherosclerosis and can be detected before structural changes to the vessel wall are apparent on angiography or ultrasound [7]. It has already been reported that hs-CRP has been shown to independently predict myocardial infarction, stroke, and peripheral artery disease [8]. Tumor necrosis factor-α, instead, is a macrophage-derived inflammatory factor that is linked to inflammation [9]. Metalloproteinases have a role as a cardiovascular risk factor; a large body of evidence asserts the role of MMPs in atherosclerosis. Both MMP-2 and MMP-9 are synthesized and secreted locally in atherosclerotic lesions, predominantly by monocyte derived macrophages and endothelial cells. In addition, through their proteolytic activity, MMPs are capable of degrading the fibrous cap of atherosclerotic plaque, thus contributing to plaque destabilization. We recorded a reduction on markers of endothelial damage in the group treated with mesoglycan compared to placebo. These results do not surprise considering mesoglycan mechanism of action. The reduction of MMP-2 and -9, and TNF-α by mesoglycan could have a positive role in contrasting atherosclerosis progression [10,11]. 

These positive effects of mesoglycan on endothelial damage markers have a clinical correspondence in the increase of pain free walking distance compared to placebo. The results we recorded with mesoglycan on pain free walking distance were in line with what was previously reported by Gossetti et al. [12] in a study that administered mesoglycan at the same dose of our study, but for 2 + 2 months, with a washout of two months without mesoglycan. Another positive result of this trial was the demonstration of the good drug tolerability, with no serious adverse events reported, which is in line with what was previously published by Ambrosio et al. [13] who performed laboratory tests showing no important clinical changes in safety parameters.

Even if we recorded an improvement of clinical parameters including ankle/brachial pressure index (ABI index), pain free walking distance, and transcutaneous oxygen pressure (TcPO_2_) with mesoglycan compared to placebo, these data should be take very cautiously, given the small number of patients involved, and also considering that the symptomless were self-reported. 

Of course our study has some limitations: the short observational period, and the fact that we used pain free walking distance as parameter to assess walking ability; some evidence from previous studies considered this parameter not reliable enough, but we used it because it was easy to measure, and it was determined with a non-invasive method.

## 4. Material and Methods

### 4.1. Study Design

This randomized, placebo controlled trial was conducted at the Department of Internal Medicine and Therapeutics, University of Pavia (Pavia, Italy). The study protocol was approved by the institutional review board and was conducted in accordance with the Declaration of Helsinki and its amendments.

Suitable patients, identified from the review of case notes and/or computerized clinic registers, were contacted by the investigators in person or by telephone. All patients provided written informed consent to participate. 

### 4.2. Patients 

We enrolled 64 Caucasian, aged ≥18 of either sex, type 2 diabetic patients according to the ESC (European Society of Cardiology) and EASD (European Association for the Study of Diabetes) Guidelines criteria [14], who were affected by peripheral artery disease according to the Society for Vascular Surgery Lower Extremity Guidelines [15]; in particular, we considered the initial time to onset of claudication as diagnostic criteria. 

We included patients with stage IIa peripheral artery disease, according to Leriche-Fontaine classification. Patients were excluded if they had a history of ketoacidosis or had unstable or rapidly progressive diabetic retinopathy, nephropathy, or neuropathy; impaired hepatic function (defined as plasma aminotransferase and/or γ-glutamyltransferase level higher than three times the upper limit of normal (ULN) for age and sex), impaired renal function (defined as serum creatinine level higher than the ULN for age and sex), or severe anemia. Patients with serious cardiovascular disease (CVD) (e.g., New York Heart Association class I–IV congestive heart failure or a history of myocardial infarction or stroke) or cerebrovascular conditions within six months before study enrolment also were excluded. Women who were pregnant or breastfeeding or of childbearing potential and not taking adequate contraceptive precautions were also excluded.

### 4.3. Treatments 

The patients fulfilling the inclusion and exclusion criteria were randomized with a 1:1 ratio, to mesoglycan (Prisma^®^, Mediolanum Farmaceutici Spa, Milano, Italy), 50 mg twice a day, or placebo (Mediolanum Farmaceutici Spa Milano, Italy), taken far from meals, for six months. Both mesoglycan and placebo were supplied as identical, opaque, white capsules in coded bottles to ensure the blind status of the study. Randomization was done using a drawing of envelopes containing randomization codes prepared by a statistician. A copy of the code was provided only to the responsible person performing the statistical analysis. The code was only broken after database lock, but could have been broken for individual subjects in cases of an emergency. Medication compliance was assessed by counting the number of pills returned at the time of specified clinic visits. At baseline, we weighed participants and gave them a bottle containing a supply of the study medication for at least 100 days. Throughout the study, we instructed patients to take their first dose of new medication on the day after they were given the study medication. At the same time, all unused medication was retrieved for inventory. Compliance assessment was performed every three months. All medications were provided free of charge.

### 4.4. Diet 

Subjects began a controlled-energy diet (near 600 kcal daily deficit) based on American Heart Association (AHA) recommendations [16] that included 50% of calories from carbohydrates, 30% from fat (6% saturated), and 20% from proteins, with a maximum cholesterol content of 300 mg/day and 35 g/day of fiber. Patients were not treated with vitamins or mineral preparations during the study.

Standard diet advice was given by a dietitian and/or specialist doctor. A dietitian and/or specialist doctor periodically provided instruction on dietary intake recording procedures as part of a behavior modification program and then later used the subject’s food diaries for counselling. 

### 4.5. Assessments 

Before starting the study, all patients underwent an initial screening assessment that included a medical history, physical examination, vital signs, and a 12-lead electrocardiogram. We evaluated at the baseline, and after six months these parameters: body weight, glycated hemoglobin, fasting and post-prandial glucose, levels of some markers of endothelial damages including metalloproteinase-2 and -9 (MMP-2 and -9), sICAM-1, sVCAM-1, IL-6, sE-selectin, hs-CRP, TNF-α, and plasminogen activator inhibitor-1 (PAI-1). 

At baseline, and after six months, we also evaluated: the pain free walking distance, the ankle/brachial index, and the O_2_ tension, which was evaluated via a cutaneous oximetry technique. 

For a description of how various parameters were assessed, please see our previous papers [17,18,19]. 

Regarding the ABI index, it was measured in both the dorsalis pedis and the posterior tibial vessels, with the patient supine and temperature acclimatized. The highest of these measurements was used to calculate the ankle brachial pressure index, according to the Society for Vascular Surgery Lower Extremity Guidelines [15]. The most affected limb was considered for ABI calculation. 

Tissue perfusion was estimated by measuring the transcutaneous oxygen pressure with an electrode attached to the foot after local heating to induce maximum vasodilation. We used PeriFlux 6000 (Perimed AB, Järfälla-Stockholm, Sweden). The temperature of all probes was 44.5 °C, to allow maximal local vasodilatation, thereby decreasing the arterial to skin pressure gradient, according to what was previously reported by Urban et al. [20].

The pain-free walking distance was assessed on a treadmill set at 40 m/min with a 10% incline until pain forced patients to stop, according to Gardner et al. [21]. 

### 4.6. Statistical Analysis 

An intention-to-treat analysis was conducted in patients who had received ≥1 dose of study medication and had a subsequent efficacy observation. Patients were included in the tolerability analysis if they had received ≥1 dose of trial medication and had undergone a subsequent tolerability observation. Continuous variables were tested using a two-way repeated measures analysis of variance (ANOVA) to assess overall differences. Intervention effects were adjusted for additional potential confounders using analysis of covariance (ANCOVA). ANOVA was also used to assess the significance within and between groups. The statistical significance of the independent effects of treatments on the other variables was determined using ANCOVA. A one-sample *t* test was used to compare values obtained before and after treatment administration; two-sample *t* tests were used for between-group comparisons [22]. Statistical analysis of data was performed using the Statistical Package for Social Sciences software version 11.0 (SPSS Inc., Chicago, IL, USA). Data are presented as mean ± standard deviation (SD). For all statistical analyses, *p* < 0.05 was considered statistically significant.

## 5. Conclusions

Our study showed that supplementation with mesoglycan improved endothelial dysfunction in type 2 diabetic patients with peripheral artery disease. Regarding the preliminary data suggesting also a slight improvement of clinical parameters such as pain free walking distance, more data and a bigger sample of patients are necessary to better verify this aspect. 

## Figures and Tables

**Table 1:** Results at baseline and at the end of the study regarding biochemical parameters.

Parameters	Mesoglycan		Placebo	
	Baseline	Six Months	Baseline	Six Months
*N*	33	31	31	28
Sex (M/F)	16/17	15/16	17/14	15/13
Smokers (M/F)	10/7	9/7	11/8	11/8
Age (years)	64.2 ± 5.7	-	63.2 ± 5.3	-
Diabetes duration (years)	7.3 ± 3.7	-	7.4 ± 3.9	-
Weight (kg)	79.9 ± 3.0	79.2 ± 2.8	78.3 ± 2.2	78.6 ± 2.4
Height (m)	1.70 ± 0.04	-	1.69 ± 0.03	-
BMI (kg/m^2^)	27.6 ± 1.1	27.4 ± 0.9	27.4 ± 1.0	27.5 ± 1.1
FPG (mg/dL)	126.3 ± 12.7	122.5 ± 10.4	127.5 ± 12.9	121.4 ± 9.9
PPG (mg/dL)	150.4 ± 17.9	147.2 ± 16.5	153.4 ± 18.1	148.6 ± 16.9
HbA_1c_ (%)	7.5 ± 0.6	7.3 ± 0.5	7.3 ± 0.5	7.2 ± 0.4
MMP-2 (ng/mL)	1252.7 ± 136.5	978.1 ± 97.1 *^,^^‡^	1254.5 ± 137.1	1189.2 ± 112.8
MMP-9 (ng/mL)	480.4 ± 55.9	381.8 ± 34.2 *^,^^‡^	475.2 ± 53.3	433.2 ± 48.1
sICAM-1 (ng/mL)	204.6 ± 14.1	161.2 ± 8.0 *	198.3 ± 13.0	180.2 ± 8.9
sVCAM-1 (ng/mL)	641.9 ± 177.5	504.2 ± 114.2 ^†^^,^^‡^	645.2 ± 180.2	627.2 ± 155.2
IL-6 (pg/mL)	3.8 ± 2.3	2.7 ± 1.9 ^†^^,^^‡^	3.6 ± 2.2	3.3 ± 2.0
hs-CRP (mg/L)	2.3 ± 1.1	1.7 ± 0.5 *	2.0 ± 0.9	1.9 ± 0.8
sE-selectin (ng/mL)	32.8 ± 6.1	24.2 ± 3.8 *^,^^‡^	30.5 ± 5.4	29.2 ± 4.5
TNF-α (ng/mL)	2.1 ± 1.0	1.5 ± 0.6 *^,^^‡^	2.2 ± 1.1	1.9 ± 0.9
PAI-1 (ng/mL)	36.4 ± 9.5	32.5 ± 8.3	41.3 ± 10.6	40.3 ± 10.0

Data are expressed as mean ± standard deviation; * *p* < 0.05 vs. baseline; ^†^ *p* < 0.01 vs. baseline; ^‡^ *p* < 0.05 vs. placebo; BMI: body mass index; HbA_1c_: glycated hemoglobin; FPG: fasting plasma glucose; PPG: post-prandial plasma glucose; MMP-2: metalloproteinase-2; MMP-9: metalloproteinase-9; sICAM-1: soluble intercellular adhesion molecule-1; sVCAM-1: soluble vascular cell adhesion protein-1; IL-6: interleukin-6; sE-selectin: soluble selectin-E; hs-CRP: high sensitivity C-reactive protein; TNF-α: tumor necrosis factor-α; PAI-1: plasminogen activator inhibitor-1.

**Table 2:** **C**linical parameter results at baseline and at the end of the study.

Parameters	Mesoglycan		Placebo	
	Baseline	Six Months	Baseline	Six Months
Walking distance pain free (m) for the 18 patients improved	247.2 ± 20.3	383.4 ± 41.2 ^†,‡^	249.2 ± 21.7	255.1 ± 23.3
Walking distance pain free (m) for patients not improved)	254.3 ± 22.8	269.8 ± 25.7		
Ankle/brachial index (most affected limb) for the 18 patients improved	0.57 ± 0.16	0.80 ± 0.31 *^,‡^	0.61 ± 0.19	0.56 ± 0.17
Ankle/brachial index (most affected limb) for patients not improved	0.58 ± 0.17	0.70 ± 0.25		
TcPO_2_ (mmHg) for the 18 patients improved	44.2 ± 11.3	56.1 ± 15.2 *^,‡^	41.3 ± 10.7	39.1 ± 9.9
TcPO_2_ (mmHg) for patients not improved	40.5 ± 10.9	47.9 ± 13.8		

Data are expressed as mean ± standard deviation; * *p* < 0.05 vs. baseline; ^†^ *p* < 0.01 vs. baseline; ^‡^ *p* < 0.05 vs. placebo; TcPO_2_: transcutaneous oxygen pressure.

**Table 3:** Concomitant medications.

Parameters	Mesoglycan	Placebo
*N*	33	31
Anti-diabetic drugs	33 (100)	31 (100)
Metformin	25 (75.8)	24 (77.4)
Sulphonylureas	8 (24.2)	5 (16.2)
Meglitinides derivatives	3 (9.1)	2 (6.5)
α-glucosidase inhibitors	4 (12.1)	5 (16.1)
Thiazolidinediones	3 (9.1)	3 (9.7)
DPP-4 inhibitors	6 (18.2)	5 (16.1)
GLP-1 analogues	2 (2.3)	3 (9.7)
Insulin	4 (12.1)	2 (6.5)
Anti-aggregants	33 (100)	31 (100)
ASA	21 (63.6)	23 (74.2)
Ticlopidine	3 (9.1)	2 (6.5)
Clopidogrel	9 (27.3)	6 (19.3)

Data are *n* (%); ASA: acetylsalicylic acid; DPP-4 inhibitors: dipeptidyl peptidase-4 inhibitors; GLP-1 analogues: glucagon-like peptide-1 analogues.
